# Impact of Treat-to-Target Therapy on Bone Mineral Density Loss in Patients With Rheumatoid Arthritis: A Prospective Cohort Study

**DOI:** 10.3389/fendo.2022.867610

**Published:** 2022-05-17

**Authors:** Hong Huang, Yu Wang, Wenhui Xie, Yan Geng, Dai Gao, Zhuoli Zhang

**Affiliations:** Department of Rheumatology and Clinical Immunology, Peking University First Hospital, Beijing, China

**Keywords:** rheumatoid arthritis, treat-to-target strategy, disease activity, bone mineral density, osteoporosis

## Abstract

**Background:**

Osteoporosis is a common comorbidity of rheumatoid arthritis (RA). Although RA disease activity has been demonstrated to be associated with bone loss in previous studies, most of them were cross-sectional studies and not in the context of treat-to-target (T2T) strategies.

**Objectives:**

This study aimed to evaluate the association of disease activity with bone mineral density (BMD) changes in the context of T2T strategies in a prospective RA cohort.

**Methods:**

RA patients were enrolled from a prospective CENTRA cohort of Peking University First Hospital. The follow-ups have been scheduled every 3 to 6 months. BMD was repeated at baseline, 1 year, and then every other year. Demographics, baseline clinical features, laboratory data, and medications at each visit were recorded. Time-adjusted mean disease activity scores were adopted to reflect the overall disease activity during follow-up. The influence of univariable associations between predictors and BMD was investigated using linear regression.

**Results:**

A total of 268 patients were included in our analysis. Their mean age was 50 (12.9) years, and 224 (83.6%) were women. The median (IQR) disease duration was 48.7 (107.6) months. Osteoporosis in the lumbar spine was observed in 23.1% of patients and 9.3% in the femoral neck at enrollment. Older age, higher SDAI score, and lower BMI were associated with osteoporosis at baseline. The proportion of patients who achieved DAS28-ESR, CDAI, and SDAI remission or LDA at the end of the first year was 71.5%, 68.8%, and 67.4%, respectively. Reevaluations of BMD at 1 year were applied to 144 patients. Mean decreases of BMDs were 1.75% at the lumbar spine and 1.40% at the femoral neck at 1 year from baseline, respectively. Patients who achieved remission had less yearly bone loss in the lumbar spine (*p* = 0.036). Female gender was identified as a risk factor in the multiple linear regression analyses, and lower disease activity and bisphosphonates were protective factors of continuous bone loss.

**Conclusion:**

Disease activity is associated with bone loss in RA patients in the context of T2T strategies, and those who achieved remission had less yearly bone loss.

## 1 Introduction

Rheumatoid arthritis (RA) is a chronic inflammatory disease affecting about 1% of the general population worldwide (1, 2). RA is characterized by synovial inflammation and proliferation, accompanied by cartilage erosion and bone loss (3). Therefore, osteoporosis (OP) is one of the most common comorbidities of RA, which may lead to fragility fractures (4).

OP was reported in 15%–36% of RA patients depending on different measuring sites and races (5–9), and fragility fracture was about 2-fold in patients with OP secondary to RA compared with the general population (10). Moreover, in the Fracture Risk Assessment Tool proposed by the World Health Organization (WHO) aiming to evaluate the 10-year probability of hip and major osteoporotic fractures, RA is the only disease included as a risk factor (11).

Although previous studies have demonstrated the deleterious influence of active disease on bone loss (6, 12–14), most of these studies were cross-sectional designs or conducted before the introduction of treat-to-target (T2T) strategies. A recent randomized controlled trial aiming to discover the effect of T2T strategies on bone erosion progression found that achieving sustained remission defined by simplified disease activity score (SDAI) was associated with partial erosion repair (15). Biological disease-modifying antirheumatic drugs (bDMARDs) and targeted synthetic disease-modifying antirheumatic drugs (tsDMARDs) have been demonstrated to halt the progression of bone erosions, even partially repair erosion, and protect from bone loss in patients with RA (16–19). A recent study found that when RA disease activity is well controlled using the T2T strategy, the risk of bone mineral density (BMD) loss is diminished, suggesting a correlation between RA disease activity and bone loss (20). However, it has not clearly demonstrated the relationship between disease activity and bone loss dynamically. Until now, there is little evidence clarifying the influence of the T2T strategy on BMD and its influential factors. Therefore, we set out to evaluate the changes in BMD in our prospective RA cohort in the context of T2T strategies.

## 2 Materials and Methods

### 2.1 Study Design

The Collaboratively intENsive Treat-to-target in RA (CENTRA) cohort is a prospective observational real-world cohort conducted in the Rheumatology and Clinical Immunology Department of Peking University First Hospital since October 2015. More details of the cohort have been described in our previous study (21).

### 2.2 Participants

Inclusion criteria:
1. ≥18 years of age.
2. Fulfilled the 2010 American College of Rheumatology/European League Against Rheumatism classification criteria for RA (22).
Exclusion criteria:
1. Patients with comorbidities of thyroid disease with abnormal thyroid function or parathyroid diseases, poorly controlled diabetes mellitus, malabsorption, chronic diarrhea, and celiac disease.
2. Patients treated with menopausal hormone therapies.

### 2.3 Data Collection

#### 2.3.1 Baseline Demographics

Age, sex, RA disease duration, body mass index (BMI), current and past smoking status, menopausal status, and past medical history were recorded. Menopause was defined as a permanent end of menstrual bleeding monthly periods for at least 1 year.

#### 2.3.2 Laboratory Results at Each Visit

Complete blood count, hepatic and renal function, fasting glucose, erythrocyte sedimentation rate (ESR, mm/h), C-reactive protein (CRP, mg/L), and rheumatoid factor (RF, IU/ml), are routinely tested at each visit. Anticyclic citrullinated peptides (anti-CCP, RU/ml) and anti-mutated citrullinated vimentin (anti-MCV, U/ml) were tested at baseline, year 1, and then at 2-year intervals.

#### 2.3.3 Disease-Related Clinical Data at Each Visit

Tender joint count (TJC) and swollen joint count (SJC) based on 28 joints, patient’s global assessment (PGA, 100 mm VAS), evaluator’s global assessment (EGA, 100 mm VAS), and questionnaires concerning patient-reported outcomes are performed at baseline and all visits of follow-up. Disease activity scores based on the 28-joint count and ESR (DAS28-ESR), the simplified disease activity index (SDAI), and the clinical disease activity index (CDAI) are calculated to assess the disease activity. The formula used to calculate the composite disease activity scores with corresponding definitions of remission and LDA are as follows (23–25):


CDAI=(TJC28+SJC28+PGA+EGA)≤2.8(remission);>2.8and≤10(LDA)

SDAI=(TJC28+SJC28+CRP+PGA+EGA)≤3.3(remission);3.3and≤11(LDA)

$$DAS28-ESR=\left(0.56\sqrt{}\left[TJC28\right]+0.28\sqrt{}\left[SJC28\right]+\text{ln}\;[ESR]+0.014\times PGA\right)<2.6\left(remission\right);\geq 2.6and\leq 3.2\left(\mathrm{LDA}\right)$$

To evaluate the overall disease activity during follow-up, more objective, time-adjusted mean (AM) ESR, CRP, and disease activity scores were adopted to eliminate the interference of varying time intervals. The area under the curve of ESR, CRP, or disease activity scores over time was calculated by adding the area of each of the blocks of visit interval and then dividing them by the length of time for the whole period (26, 27).


$$\frac{\sum_{i=2}^{n}\;(\frac{X_{i}+X_{i-1}}{2})t_{i}}{\sum_{i=2}^{n}\;t_{i}}$$

*X_i_* = DAS28(ESR) of number *i* follow-up, *t_i_* = the time interval of number *i* and number *i*–1 follow-up.

#### 2.3.4 Assessment of OP and Fractures

BMDs at the femoral neck and lumbar spine were measured at baseline, first year, and then every other year by dual-energy X-ray absorptiometry (DXA) using the Norland DXA scanner. BMDs were expressed in absolute values as grams of mineral content per square centimeters of bone area (g/cm^2^) and T-score. The percentage of change in BMD (%ΔBMD) was calculated.

According to the WHO classification criteria, OP was defined as a BMD that lies 2.5 standard deviations (SD) or more below the average value (a T-score of <−2.5 SD) (28). Identification of fracture was based on patient-reported symptoms and subsequent confirmation by radiography.

The primary endpoint was the percentage of change in lumbar BMD and femoral neck BMD from baseline. The first occurrence of a fracture was the secondary endpoint of the study.

#### 2.3.5 Medications at Each Visit

Medications at baseline including conventional synthetic disease-modifying antirheumatic drugs (csDMARDs), for instance, methotrexate (MTX), leflunomide (LEF), hydroxychloroquine (HCQ), sulfasalazine (SSZ), glucocorticosteroids (GC), as well as biological/targeted synthetic disease-modifying antirheumatic drugs (DMARDs) (b/tsDMARDs) were recorded. The prescribed GC was converted to prednisolone equivalent dose and collected as a cumulative dose during follow-up.

### 2.4 Statistical Analysis

The descriptive statistics contained means and standard deviation (SD), medians and interquartile ranges (IQR), or percentages for each relevant variable. The difference between the two groups was assessed by the *t*-test or nonparametric test. A comparison of categorical data was performed using the Chi-square test. A logistic regression model with odds ratio (OR) and 95% confidence interval (CI) was used to determine the variables associated with OP at baseline.

The influence of univariable associations between predictors and BMD was investigated using linear regression analysis. To identify independent predictors of BMD, we performed stepwise multivariable linear regression analyses that included variables being significant in the univariable analyses of the total sample at *p* < 0.10.

All reported *p*-values were 2-sided, and only associations with a *p* < 0.05 were considered statistically significant. All statistical analyses were performed using SPSS software (version 22, IBM Corporation, Armonk, USA).

## 3 Results

### 3.1 Demographics and Baseline Clinical Characteristics of RA Patients

A total of 268 consecutive patients contributing to 1,842 clinical visits were included in the study. Their mean (SD) age was 50 (12.9) years, with a median (IQR) disease duration of 48.7 (107.6) months. Of these patients, 224 (83.6%) were women and half of them (112, 50%) were postmenopausal. The median (IQR) age of menopause and the time for menopause to baseline were 50 (5.5) years and 7.84 (8.6) years, respectively. The median (IQR) serum creatinine was 70 (15) μmol/L and the mean (SD) eGRF was 89.33 (16) ml/min/1.73 m^2^. Median (IQR) fasting glucose was 5.14 (0.7) mmol/L. At enrollment, the mean (SD) DAS28-ESR was 3.4 (1.5), and the median (IQR) CDAI and SDIA were 10 (14.45) and 10.30 (16.53), respectively. The demographics and baseline clinical characteristics of RA are shown in **Table 1**.

**Table 1:** Baseline characteristics of 268 RA patients and those repeated BMD at 1 year.

	All patients (*n* = 268)	Repeated BMD at 1 year (*n* = 144)
Women (*n* (%))	224 (83.6)	120 (83.3)
Age (mean (SD), year)	50.0 (12.9)	50.3 (13.6)
Age of onset (mean (SD), year)	43.0 (14.0)	43.6 (14.0)
BMI (mean (SD), kg/m^2^)	22.9 (3.9)	23.1 (4.0)
Postmenopause (*n* (%))	112 (50.0)^a^	60 (50.0)^b^
Age of menopause (median (IQR), year)	50 (5.5)^c^	50 (4.8)^d^
Time since menopause (median (IQR), year)	7.84 (8.6)^c^	9 (9.7)^d^
Current or past smoking (*n* (%))	38 (14.2)	20 (13.9)
History of fracture (*n* (%))	32 (11.9)	17 (11.8)
Family history of fracture (*n* (%))	37 (13.8)	24 (16.7)
Current or past glucocorticoids (*n* (%))	90 (33.6%)	59 (40.9%)
Current or past bisphosphonates (*n* (%))	5 (1.9%)	28 (19.4%)
Disease duration (median (IQR), month)	48.7 (107.6)	47.9 (95.9)
Serum creatinine (median (IQR), μmol/L)	70 (15)	73 (14.8)
eGRF (mean (SD), ml/min/1.73 m^2^)	89.33 (16)	87.39 (17.3)
Fasting glucose (median (IQR), mmol/L)	5.14 (0.7)	5.18 (0.8)
ESR (median (IQR), mm/h)	21.0 (30.0)	20.5 (29.0)
CRP (median (IQR), mg/L)	5.8 (11.2)	6.2 (10.8)
SJC (median (IQR))	1.0 (3.0)	1.0 (3.0)
TJC (median (IQR))	2.0 (5.0)	2.0 (4.0)
PGA (0–100) (median (IQR))	30.0 (33.8)	30 (30)
EGA (0–100) (median (IQR))	20.0 (30.0)	20.0 (20.0)
DAS28 (mean (SD))	3.4 (1.5)	3.4 (2.5)
SDAI (median (IQR))	10.3 (16.5)	9.3 (12.2)
CDAI (median (IQR))	10 (14.5)	8.0 (11.6)
RF (median (IQR))	85.8 (246.6)	83.2 (260.8)
Anti-CCP (median (IQR))	116.0 (159)	117 (169.3)
HAQ score (median (IQR))	4.0 (13.5)	3.0 (10.75)

Values are presented as mean (SD) or median (IQR), as applicable.

SD, standard deviation; IQR, interquartile ranges.

a In 224 female patients.

b In 120 female patients.

c In 112 postmenopausal female patients.

d In 60 postmenopausal female patients.

### 3.2 Frequency and Risk Factors of OP at Baseline

There were 62 (23.1%) patients with OP at the lumbar spine and 25 (9.3%) patients with OP at the femoral neck at baseline. Compared with those non-OP patients, patients with OP at the lumbar spine were significantly older (58 vs. 47.6, *p* < 0.001), more likely to be postmenopausal (84.9% vs. 39.2%, *p* < 0.001), and had lower BMI (21.3 vs. 23.4, *p* < 0.001) and higher disease activity score. Besides, more OP patients ever exposed to GC in the past (46.8% vs. 29.6%, *p* = 0.014). While at the femoral neck, we found significant associations of OP with gender (*p* < 0.001), age (*p* < 0.001), postmenopause (*p* < 0.001), BMI (*p* = 0.019), and past/current smokers (*p* < 0.001) (**Table 2**).

**Table 2:** Comparisons between RA patients with OP and without OP at baseline.

	Lumbar spine			Femoral neck		
	OP (*n* = 62)	Non-OP (*n* = 206)	*p*-value	OP (*n* = 25)	Non-OP (*n* = 243)	*p*-value
Women (*n* (%))	53 (85.5)	171 (83)	0.701	10 (40)	214 (88.1)	<0.001
Age at baseline (mean (SD), year)	58 (9.6)	47.6 (12.9)	<0.001	59 (8.7)	49.1 (13)	<0.001
Age of onset (mean (SD), year)	50.1 (12.8)	40.9 (13.7)	<0.001	49.2 (13.1)	42.4 (14)	0.021
BMI (mean (SD), kg/m^2^)	21.3 (3.5)	23.4 (3.8)	<0.001	21.2 (3.8)	23.1 (3.8)	0.019
Postmenopause^a^ (*n* (%))	45 (84.9)	67 (39.2)	<0.001	9 (90)	103 (48.1)	<0.001
Current or past smoking (*n* (%))	8 (12.9)	30 (14.6)	0.838	13 (52)	25 (10.3)	<0.001
History of fracture (*n* (%))	9 (14.5)	23 (11.2)	0.504	6 (24)	26 (10.7)	0.103
Past glucocorticoids (*n* (%))						
Family history of fracture (*n* (%))	5 (8.1)	32 (15.5)	0.148	3 (12)	34 (14)	1.000
History of glucocorticoids (*n* (%))	29 (46.8)	61 (29.6)	0.014	9 (36)	81 (33.3)	0.474
History of bisphosphonates (*n* (%))	3 (4.8)	2 (1)	0.150	0 (0)	5 (2.1)	0.469
Disease duration (mean (SD), month)	95.7 (103.5)	81.5 (98.4)	0.324	119.6 (133.5)	81.2 (95.1)	0.173
ESR (median (IQR), mm/h)	25.5 (31.7)	18 (25)	0.002	30 (35)	20 (27)	0.281
CRP (median (IQR), mg/L)	9 (21.1)	5.4 (9.3)	0.016	8.2 (19.5)	5.8 (10.8)	0.205
TJC (median (IQR))	3 (5.3)	2 (5)	0.037	2 (5)	2 (5)	0.638
SJC (median (IQR))	1.5 (5)	1 (3)	0.026	0 (3)	1 (3)	0.402
PGA (0–100) (median (IQR))	30 (40)	30 (40)	0.069	25 (30)	30 (35)	0.441
EGA (0–100) (median (IQR))	30 (35)	20 (30)	0.025	20 (42.5)	20 (30)	0.972
HAQ score (median (IQR))	0.4 (0.8)	0.1 (0.6)	<0.001	7 (28)	3 (13)	0.103
DAS28 (mean (SD))	4.2 (1.8)	3.5 (1.6)	0.004	3.6 (2)	3.7 (1.7)	0.852
CDAI (median (IQR))	12 (16.5)	8.3 (13)	0.014	8 (15.5)	10 (14.5)	0.639
SDAI (median (IQR))	14.6 (19.1)	9.2 (14.6)	0.008	8.6 (18.1)	10.3 (16.5)	0.802
RF (median (IQR), mg/L)	67.2 (224.7)	93.8 (260)	0.208	57 (166)	90.5 (249.5)	0.151
Anti-CCP (median (IQR), RU/ml)	141 (148)	98 (163)	0.171	69 (153)	119 (158)	0.254

Values are presented as mean (SD) or median (IQR), as applicable.

SD, standard deviation; IQR, interquartile ranges.

a The total numbers were 53 for OP group and 171 for non-OP group at the lumbar spine; and the total numbers were 10 for OP group and 214 for non-OP group at the femoral neck.

The following variables were included in the multivariable models: age, female gender, postmenopausal, BMI, HAQ score, and SDAI score at baseline based on the results of univariate logistic regressions (**Table 3**). Stepwise multivariable linear regression analysis revealed that the most significant risk factor for OP was older age (at the lumbar spine: OR, 1.12 (95% CI, 1.08–1.16), *p* < 0.001; at the femoral neck: OR, 1.11 (95% CI, 1.04 = 1.20), *p* = 0.004). A higher SDAI score was identified as a risk factor for OP at the lumbar spine only (OR, 1.034 (95% CI, 1.01–1.06), *p* = 0.019), while higher BMI seemed to be a protective factor at the lumbar spine (OR, 0.839 (95% CI, 0.752–0.937), *p* = 0.002).

**Table 3:** Univariate logistic regression model on OP at different measured sites at baseline.

	Lumbar spine			Femoral neck		
	*β*	*p*-value	OR (95% CI)	*β*	*p*-value	OR (95% CI)
Traditional risk factors						
Age at baseline	0.075	<0.001	1.08 (1.05, 1.11)	0.070	<0.001	1.07 (1.03, 1.12)
Age of onset	0.050	<0.001	1.05 (1.03, 1.08)	0.035	0.024	1.04 (1.01, 1.07)
Women	0.187	0.645	1.21 (0.54, 2.67)	−2.404	<0.001	0.09 (0.04, 0.22)
Postmenopausal	2.167	<0.001	8.73 (3.88, 19.67)	2.272	0.033	9.70 (1.21, 77.89)
BMI	−0.158	<0.001	0.85 (0.78, 0.93)	−0.147	0.020	0.86 (0.76, 0.98)
RA-related risk factors						
HAQ	0.656	0.001	1.93 (1.30, 2.86)	0.030	0.022	1.03 (1.00, 1.06)
DAS28	0.240	0.005	1.27 (1.08, 1.50)	−0.023	0.851	0.98 (0.77, 1.25)
SDAI	0.027	0.007	1.03 (1.01, 1.05)	0.004	0.805	0.97 (0.97, 1.03)

### 3.3 Disease Activity of Patients During Follow-Up

**Figure 1:**
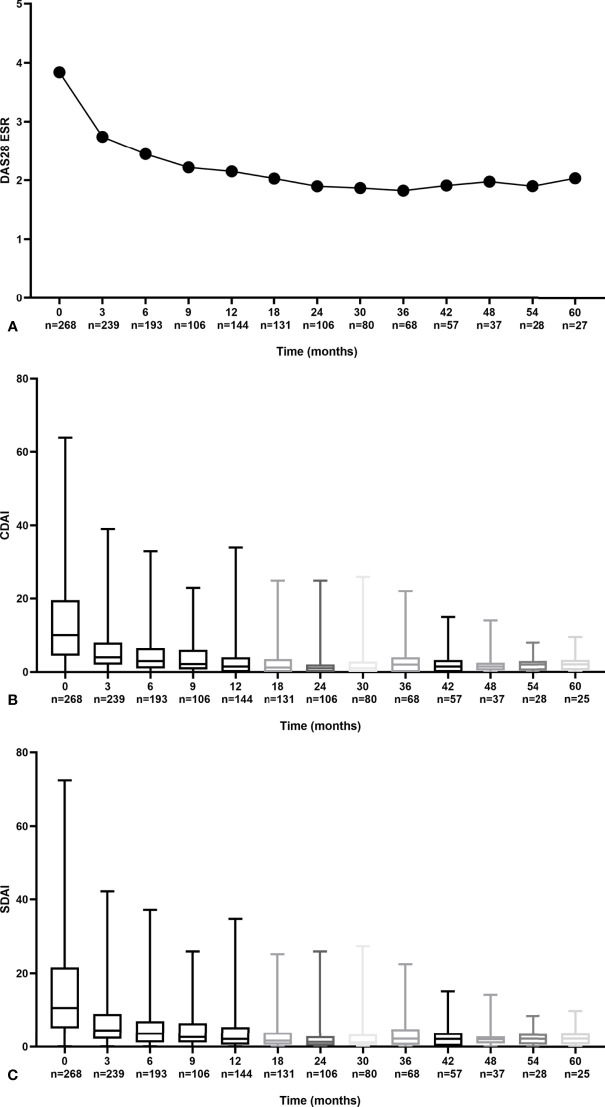
Trends in disease activity scores during follow-up by DAS28 **(A)** CDAI **(B)** and SDAI **(C)**.

Overall, a steady decrease in disease activity based on the time-adjusted mean DAS28, CDAI, and SDAI throughout the follow-up year was observed (**Figure 1**). For DAS28, a dramatic decline of 0.93 (25.9%) at the end of 3 months and 1.44 (40.1%) at the end of 1 year from baseline was observed. Similarly, CDAI and SDAI dramatically dropped 5.11~5.25 (54.8~63.6%) in the first 3 months and 6.75~7.22 (77.5~81.8%) at the end of 1 year.

**Figure 2:**
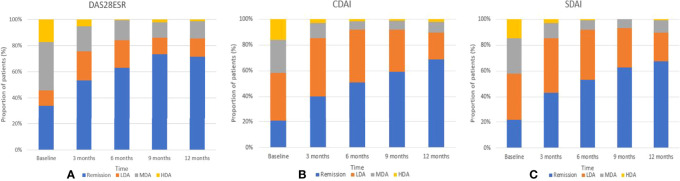
Trends in the percentage of remission, low, moderate, and high disease activity over the first years by DAS28 **(A)** CDAI **(B)** and SDAI **(C)**.

At the end of the first year, the mean (SD) DAS28-ESR was 2.15 (1.09), and the median (IQR) CDAI and SDIA were 1.50 (4.00) and 2.10 (4.52), respectively. The proportion of patients who achieved DAS28-ESR, CDAI, and SDAI remission was 71.5%, 68.8%, and 67.4% (**Figure 2**). Similar trends of declined disease activity were observed in a 3-year follow-up (**Supplementary Figure S1**). At the end of 3 years, the mean (SD) DAS28-ESR was 1.82 (0.78), and the median (IQR) CDAI and SDIA were 1.00 (2.00) and 1.32 (2.00), respectively.

### 3.4 The Change of BMDs During Follow-Up in the Context of T2T Strategies for RA

**Figure 3:**
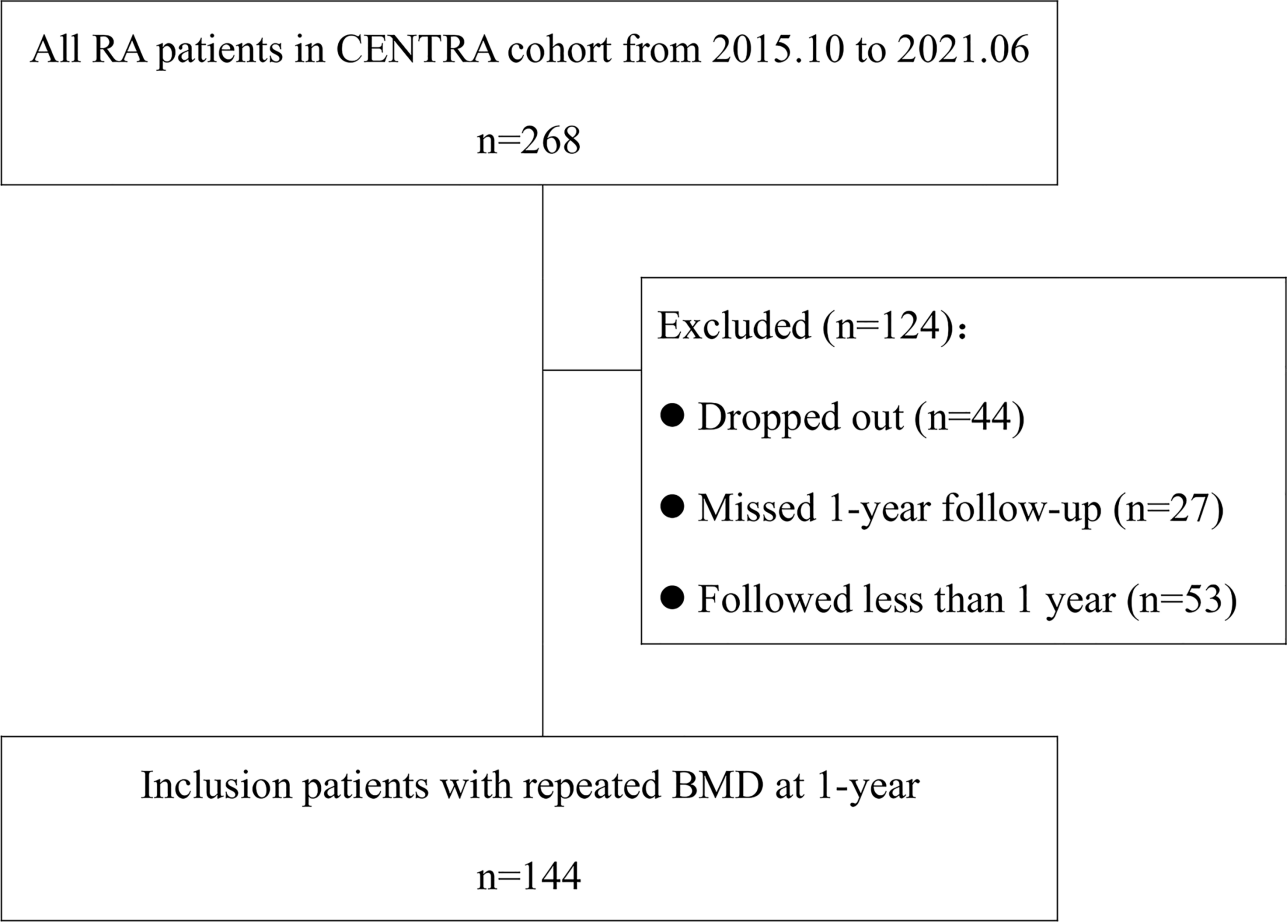
Flowchart of the study.

Among 268 patients in the CENTRA cohort, 53 were not included in the study due to a follow-up duration of less than 1 year. Because of the pandemic, 44 patients dropped out of the cohort, and 27 patients missed assessment at a 1-year time point of follow-up. The remaining 144 patients were included in the final analysis (**Figure 3**; **Table 1**). At baseline, their mean (SD) age was 50.3 (13.6) years, with a median (IQR) disease duration of 47.9 (95.9) months. Of these patients, 120 (83.3%) were women and half were postmenopausal. The mean (SD) DAS28-ESR was 3.4 (2.5), and the median (IQR) CDAI and SDIA were 8 (11.6) and 9.3 (12.2), respectively. At the end of the first year of follow-up, OP was observed in 6.9% (10/144) of patients at the lumbar spine and 10.4% (15/144) of patients at the femoral neck. At the end of 3 years, 67 patients repeated BMD. OP was observed in 9% (6/67) at the lumbar spine and 14.9% (10/67) of patients at the femoral neck. No fracture occurred during the whole follow-up period.

All patients received DMARD therapy, including GC, csDMARDs, and b/tsDMARDs, with MTX most frequently prescribed. The median (IQR) cumulative dose of prednisone was 1,522.5 (887.5) mg and 2,745 (6,437.5) mg at the end of the first year and third year, respectively. The proportions of patients who received bisphosphonate, calcium supplements, and vitamin D were 19.4% (28/144), 69.4% (100/144), and 47.9% (69/144), respectively (**Supplementary Table S1**).

Mean decreases of BMD per year were 1.75% at the lumbar spine and 1.40% at the femoral neck. Results of the univariate analyses are shown in **Table 4**. Linear associations were found between decreases in BMD with gender, history of fracture, bisphosphonate treatment, vitamin D supplement, as well as time-adjusted mean disease activity scores. Patients with higher time-adjusted DAS28 scores were more likely to have greater bone loss per year (*p* = 0.028). Similar results were observed for the disease activity measured by CDAI and SDAI. Female patients showed more bone loss than male patients. Bone mass at the lumbar spine was negatively influenced by a prior history of fracture (*p* = 0.045), although this association was also not statistically significant for the femoral neck (*p* = 0.746). Bisphosphonate treatment or vitamin D supplement decreased bone loss in our patients. GC and DMARDs seemed to be unrelated to %ΔBMD in the univariate analysis. Moreover, %ΔBMD at the femoral neck showed no such correlation in either time-adjusted DAS28 (*p* = 0.827) or other time-adjusted disease activity scores measured by CDAI or SDAI.

**Table 4:** Influence of a single factor on %ΔBMD at the lumbar spine during the first year of follow-up: by univariate regression analysis in 144 RA patients.

Variables	Lumbar spine			Femoral neck		
	*β*	95% CI	*p*-value	*β*	95% CI	*p*-value
Women	−4.114	−6.77, −1.46	0.003	−3.196	−5.37, −1.02	0.004
Postmenopausal	−0.118	−2.35, 2.11	0.917	0.432	−1.43, 2.30	0.211
History of fracture	3.195	0.08, 6.32	0.045	0.425	−2.16, 3.01	0.746
Bisphosphonate	4.980	2.50, 7.46	<0.001	1.971	−0.11, 4.06	0.064
Vitamin D supplementary	2.568	0.56, 4.58	0.013	2.043	0.41, 3.68	0.015
AMDAS28	−1.12	−2.34, −0.23	0.028	−0.101	−1.02, 0.81	0.827
AMCDAI	−0.343	−0.56, −0.13	0.002	−0.100	−0.28, 0.08	0.275
AMSDAI	−0.328	−0.53, −0.13	0.001	−0.078	−0.25, 0.09	0.361

AMDAS28, adjusted-mean disease activity scores based on 28-joint count and erythrocyte sedimentation rate; AMCDAI, adjusted-mean clinical disease activity index; AMSDAI, adjusted-mean simplified disease activity index.

**Figure 4:**
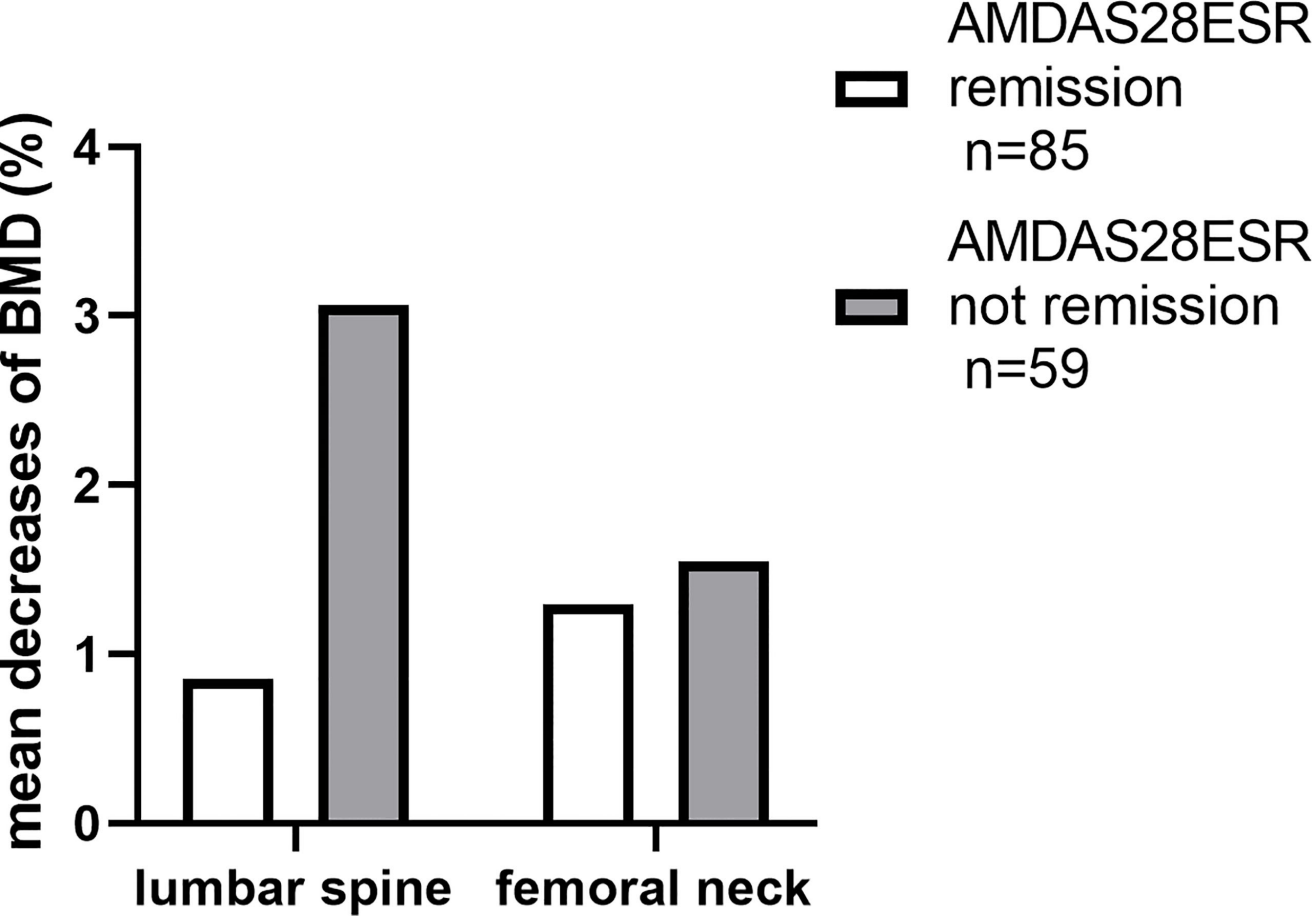
The disparities in the decreases of BMD during the first year.

To clarify the relationship between disease activity and the loss of BMD, we divided the patients into two groups according to their likelihood of achieving time-adjusted DAS28 remission (**Figure 4**). Patients who achieved remission (*n* = 85) had less yearly bone loss at the lumbar spine (*p* = 0.036).

The results of the multiple linear regression analyses are summarized in **Table 5**. Among the 144 patients, BMD loss was accelerated with increasing SDAI when gender, bisphosphonate treatment, and vitamin D supplement were also included together in the model. Female gender and bisphosphonate treatment were identified as risk and protective factors in different models, respectively. Similar results were obtained when SDAI was replaced by any other composite disease activity scores (**Supplementary Table S2**).

**Table 5:** Influence of various factors on %ΔBMD at the lumbar spine during the first year of follow-up by multiple linear regression analysis in 144 RA patients.

Variables	*β*	95% CI	*p*-value
Female	−3.060	−5.55, −0.57	0.016
Bisphosphonate supplement	4.700	2.34, 7.06	<0.001
Adjusted-mean simplified disease activity index	−0.307	−0.49, −0.12	0.001

## 4 Discussion

To the best of our knowledge, this is the first study to reveal the relationship between time-adjusted mean disease activity and BMD changes in the context of T2T strategies in a prospective RA cohort. We found that patients with lower disease activities had less bone loss.

Several studies have suggested that chronic inflammation promotes bone loss in RA patients (29–31). Moreover, the increased bone resorption and impaired bone formation caused by inflammation result in bone remodeling and increased bone fragility (32). The relationship between RA disease activity and the risk of bone loss has been demonstrated (6, 33), but the risk and protective factors for BMD loss as well as fracture were inconsistent in previous studies, mainly due to different patient selection and study design. Traditional risk factors such as older age, lower BMI, postmenopausal state, as well as disease-specific risk factors like higher disease activity were found to be related to lower BMD (12, 14, 34). By looking at various baseline clinical parameters, we found that older age, lower BMI, and higher disease activity score were related to OP in our cohort of 268 RA patients.

During 1-year follow-up in our cohort, the mean decrease of BMD was more significant in the lumbar spine than femoral neck (1.75% vs. 1.40%), and the relationship between disease activity and OP is shown in patients with low lumbar spine BMD but not low femoral neck BMD, which is similar to previous reports (14, 35). This can be explained by the different ratios of cortical to trabecular bone within the vertebra and femoral neck. The femoral neck is composed of a higher ratio of cortical bone, while the lumbar spine consists of more trabecular bone (36). Moreover, inflammation tends to increase bone turnover; therefore, the influence of RA inflammation on the lumbar spine is presumably more significant than on the femoral neck. The great values of T2T in many aspects have been demonstrated (27, 37, 38). A gradual but steady decrease in disease activity accompanied by increased target achievement has also been found in our prospective real-world cohort in the context of T2T strategy during follow-up. To better present the disease status of patients over a period, we used time-adjusted mean disease activity in our study. A linear correlation between time-adjusted mean disease activity and the change of BMD in the lumbar spine was found, indicating the key role of the T2T strategy in reducing bone loss.

The protective effect of bisphosphonate on bone loss has been significant during 1-year treatment in our cohort of RA patients. Not surprisingly, previous studies also verified the benefit of bisphosphonates on BMD (39, 40). While GC is widely considered an important risk factor for bone metabolism, nevertheless, the risk was not shown in several studies, especially in those RA patients treated with a low daily dose or low cumulative dose of GC (41–43). In the present study, we did not find a relationship between the change of BMD with GC exposure or GC cumulative dosage either. The anti-inflammatory effects of GC in favor of reducing disease activity may counterbalance its adverse effects on bone loss. The key point in determining whether the net effect of GC is detrimental or not to bone mass lies in the duration of GC exposure. In our previous TARRA cohort study, GC was used with a median time of 27 months in RA patients (44). It might be more beneficial to use GC with a short course. For DMARDs, the most important drugs for RA treatment, there is no study showing a direct correlation with the change of BMD. Our research suggests that DMARD therapy under the T2T strategies reduces bone loss *via* eliminating inflammation in RA patients.

The most important strength of the present study is that we find the relationship between disease activities and the decreases in BMD. We also acknowledge some limitations. This was a single-center study; thus, selection bias may be inevitable. Multicenter studies are warranted to confirm our findings in the future. Besides, although 268 patients were enrolled, the follow-up time was not long enough. Therefore, changes in BMD may not be obvious. Furthermore, we found no new fracture occurred during follow-up, and since fractures in our study were based on patient-reported symptoms and subsequent confirmation by radiography, the incidence might be underestimated. Finally, we did not collect the bone turnover markers in the study. We mainly focused on the changes of BMD with the control of RA disease activity, and it may be of great value to explore the changes in the bone turnover markers.

In conclusion, the female gender is overall a risk factor for OP. Persistent stable disease in the content of T2T strategies as well as bisphosphonate therapy are protective factors for bone loss in RA patients.

## Data Availability Statement

The original contributions presented in the study are included in the article/**Supplementary Material**. Further inquiries can be directed to the corresponding author.

## Ethics Statement

The studies involving human participants were reviewed and approved by the ethics committee of Peking University First Hospital. The patients/participants provided their written informed consent to participate in this study.

## Author Contributions

ZZ conceived of the study, participated in its design, and critically revised the manuscript. HH had full access to all the data collection, analysis, interpretation, and drafting of the manuscript. YW, WX, YG, and DG were study investigators and contributed to the process of data collection. All authors read and approved the final manuscript.

## Funding

This work was supported by the National Natural Science Foundation of China (No. 81771740, 81901646) and Innovation Fund for Outstanding Doctoral Candidates of Peking University Health Science Center (No. BMU2021BSS001).

## Conflict of Interest

The authors declare that the research was conducted in the absence of any commercial or financial relationships that could be construed as a potential conflict of interest.

## Publisher’s Note

All claims expressed in this article are solely those of the authors and do not necessarily represent those of their affiliated organizations, or those of the publisher, the editors and the reviewers. Any product that may be evaluated in this article, or claim that may be made by its manufacturer, is not guaranteed or endorsed by the publisher.

## References

[1] Scott DL Wolfe F Huizinga TW. Rheumatoid Arthritis. Lancet (2010) 376(9746):1094–108. doi: 10.1016/S0140-6736(10)60826-4 20870100

[2] Kvien TK Glennas A Knudsrod OG Smedstad LM Mowinckel P Forre O. The Prevalence and Severity of Rheumatoid Arthritis in Oslo. Results From a County Register and a Population Survey. Scand J Rheumatol (1997) 26(6):412–8. doi: 10.3109/03009749709065712 9433400

[3] McInnes IB Schett G. The Pathogenesis of Rheumatoid Arthritis. N Engl J Med (2011) 365(23):2205–19. doi: 10.1056/NEJMra1004965 22150039

[4] Adami G Saag KG. Osteoporosis Pathophysiology, Epidemiology, and Screening in Rheumatoid Arthritis. Curr Rheumatol Rep (2019) 21(7):34. doi: 10.1007/s11926-019-0836-7 31123839

[5] Hauser B Riches PL Wilson JF Horne AE Ralston SH. Prevalence and Clinical Prediction of Osteoporosis in a Contemporary Cohort of Patients With Rheumatoid Arthritis. Rheumatol (Oxf Engl) (2014) 53(10):1759–66. doi: 10.1093/rheumatology/keu162 24764264

[6] Dao HH Do QT Sakamoto J. Bone Mineral Density and Frequency of Osteoporosis Among Vietnamese Women With Early Rheumatoid Arthritis. Clin Rheumatol (2011) 30(10):1353–61. doi: 10.1007/s10067-011-1762-x 21547438

[7] Haugeberg G Uhlig T Falch JA Halse JI Kvien TK. Bone Mineral Density and Frequency of Osteoporosis in Female Patients With Rheumatoid Arthritis: Results From 394 Patients in the Oslo County Rheumatoid Arthritis Register. Arthritis Rheum (2000) 43(3):522–30. doi: 10.1002/1529-0131(200003)43:3<522::AID-ANR7>3.0.CO;2-Y 10728744

[8] Sinigaglia L Nervetti A Mela Q Bianchi G Del Puente A Di Munno O. A Multicenter Cross Sectional Study on Bone Mineral Density in Rheumatoid Arthritis. Italian Study Group on Bone Mass in Rheumatoid Arthritis. J Rheumatol (2000) 27(11):2582–9. 11093437

[9] Tong JJ Xu SQ Zong HX Pan MJ Teng YZ Xu JH. Prevalence and Risk Factors Associated With Vertebral Osteoporotic Fractures in Patients With Rheumatoid Arthritis. Clin Rheumatol (2020) 39(2):357–64. doi: 10.1007/s10067-019-04787-9 31691041

[10] Kim D Cho SK Choi CB Jun JB Kim TH Lee HS. Incidence and Risk Factors of Fractures in Patients With Rheumatoid Arthritis: An Asian Prospective Cohort Study. Rheumatol Int (2016) 36(9):1205–14. doi: 10.1007/s00296-016-3453-z 26965417

[11] Kanis JA McCloskey EV Johansson H Oden A Strom O Borgstrom F. Development and Use of Frax in Osteoporosis. Osteo Internat: J Establish As Res Cooperat Between Eur Foundat Osteo Natl Osteo Foundat USA (2010) 21 Suppl 2:S407–13. doi: 10.1007/s00198-010-1253-y 20464374

[12] Ahmad HA Alemao E Guo Z Iannaccone CK Frits ML Weinblatt M. Association of Low Bone Mineral Density With Anti-Citrullinated Protein Antibody Positivity and Disease Activity in Established Rheumatoid Arthritis: Findings From a Us Observational Cohort. Adv Ther (2018) 35(2):232–42. doi: 10.1007/s12325-017-0657-x PMC5818577 29368271

[13] Gough AK Lilley J Eyre S Holder RL Emery P. Generalised Bone Loss in Patients With Early Rheumatoid Arthritis. Lancet (1994) 344(8914):23–7. doi: 10.1016/s0140-6736(94)91049-9 7912297

[14] Book C Karlsson M Akesson K Jacobsson L. Disease Activity and Disability But Probably Not Glucocorticoid Treatment Predicts Loss in Bone Mineral Density in Women With Early Rheumatoid Arthritis. Scand J Rheumatol (2008) 37(4):248–54. doi: 10.1080/03009740801998747 18612924

[15] Yue J Griffith JF Xu J Xiao F Shi L Wang D. Effect of Treat-To-Target Strategies on Bone Erosion Progression in Early Rheumatoid Arthritis: An Hr-Pqct Study. Semin Arthritis Rheum (2018) 48(3):374–83. doi: 10.1016/j.semarthrit.2018.05.001 29858113

[16] Chen JF Hsu CY Yu SF Ko CH Chiu WC Lai HM. The Impact of Long-Term Biologics/Target Therapy on Bone Mineral Density in Rheumatoid Arthritis: A Propensity Score-Matched Analysis. Rheumatol (Oxf Engl) (2020) 59(9):2471–80. doi: 10.1093/rheumatology/kez655 PMC7449814 31984422

[17] Moller Dohn U Boonen A Hetland ML Hansen MS Knudsen LS Hansen A. Erosive Progression Is Minimal, But Erosion Healing Rare, in Patients With Rheumatoid Arthritis Treated With Adalimumab. A 1 Year Investigator-Initiated Follow-Up Study Using High-Resolution Computed Tomography as the Primary Outcome Measure. Ann Rheuma Dis (2009) 68(10):1585–90. doi: 10.1136/ard.2008.097048 19019887

[18] Finzel S Rech J Schmidt S Engelke K Englbrecht M Schett G. Interleukin-6 Receptor Blockade Induces Limited Repair of Bone Erosions in Rheumatoid Arthritis: A Micro Ct Study. Ann Rheuma Dis (2013) 72(3):396–400. doi: 10.1136/annrheumdis-2011-201075 22586162

[19] Lee EB Fleischmann R Hall S Wilkinson B Bradley JD Gruben D. Tofacitinib Versus Methotrexate in Rheumatoid Arthritis. N Engl J Med (2014) 370(25):2377–86. doi: 10.1056/NEJMoa1310476 24941177

[20] Yoshii I Chijiwa T Sawada N. Rheumatoid Arthritis in Tight Disease Control Is No Longer Risk of Bone Mineral Density Loss. Osteo Sarcopen (2020) 6(2):75–81. doi: 10.1016/j.afos.2020.04.002 PMC7374532 32715098

[21] Huang H Xie W Geng Y Fan Y Wang Y Zhao J. Towards a Better Implementation of Treat-To-Target Strategy in Rheumatoid Arthritis: A Comparison of Two Real-World Cohorts. Rheumatol Ther (2022) 28:1–11. doi: 10.1007/s40744-022-00441-0 PMC8960103 35347662

[22] Aletaha D Neogi T Silman AJ Funovits J Felson DT Bingham CO 3rd. Rheumatoid Arthritis Classification Criteria: An American College of Rheumatology/European League Against Rheumatism Collaborative Initiative. Arthritis Rheum (2010) 62(9):2569–81. doi: 10.1002/art.27584 20872595

[23] Smolen JS Breedveld FC Schiff MH Kalden JR Emery P Eberl G. A Simplified Disease Activity Index for Rheumatoid Arthritis for Use in Clinical Practice. Rheumatol (Oxf Engl) (2003) 42(2):244–57. doi: 10.1093/rheumatology/keg072 12595618

[24] Aletaha D Nell VP Stamm T Uffmann M Pflugbeil S Machold K. Acute Phase Reactants Add Little to Composite Disease Activity Indices for Rheumatoid Arthritis: Validation of a Clinical Activity Score. Arthritis Res Ther (2005) 7(4):R796–806. doi: 10.1186/ar1740 PMC1175030 15987481

[25] Prevoo ML van ‘t Hof MA Kuper HH van Leeuwen MA van de Putte LB van Riel PL. Modified Disease Activity Scores That Include Twenty-Eight-Joint Counts. Development and Validation in a Prospective Longitudinal Study of Patients With Rheumatoid Arthritis. Arthritis Rheum (1995) 38(1):44–8. doi: 10.1002/art.1780380107 7818570

[26] Ibanez D Urowitz MB Gladman DD. Summarizing Disease Features Over Time: I. Adjusted Mean Sledai Derivation and Application to an Index of Disease Activity in Lupus. J Rheumatol (2003) 30(9):1977–82. 12966601

[27] Xie W Li J Zhang X Li G Hao Y Zhao J. Trends in the Activity of Rheumatoid Arthritis as the Consequence of Treat-To-Target Strategy: Eight-Year Data From 2009 to 2016. Clin Exp Rheumatol (2018) 36(5):820–8. doi: 10.1136/annrheumdis-2018-eular.4050 29533754

[28] Kanis JA Melton LJ 3rd Christiansen C Johnston CC Khaltaev N. The Diagnosis of Osteoporosis. J Bone Min Res: Off J Am Soc Bone Min Res (1994) 9(8):1137–41. doi: 10.1002/jbmr.5650090802 7976495

[29] Green MJ Deodhar AA. Bone Changes in Early Rheumatoid Arthritis. Best Pract Res Clin Rheumatol (2001) 15(1):105–23. doi: 10.1053/berh.2000.0129 11358418

[30] Dougados M. Comorbidities in Rheumatoid Arthritis. Curr Opin Rheumatol (2016) 28(3):282–8. doi: 10.1097/BOR.0000000000000267 27027814

[31] Roux C. Osteoporosis in Inflammatory Joint Diseases. Osteo Internat: J Establish As Res Cooperat Between Eur Foundat Osteo Natl Osteo Foundat USA (2011) 22(2):421–33. doi: 10.1007/s00198-010-1319-x 20552328

[32] Briot K Geusens P Em Bultink I Lems WF Roux C. Inflammatory Diseases and Bone Fragility. Osteo Internat: J Establish As Res Cooperat Between Eur Foundat Osteo Natl Osteo Foundat USA (2017) 28(12):3301–14. doi: 10.1007/s00198-017-4189-7 28916915

[33] Lodder MC de Jong Z Kostense PJ Molenaar ET Staal K Voskuyl AE. Bone Mineral Density in Patients With Rheumatoid Arthritis: Relation Between Disease Severity and Low Bone Mineral Density. Ann Rheuma Dis (2004) 63(12):1576–80. doi: 10.1136/ard.2003.016253 PMC1754831 15547081

[34] Lee SG Park YE Park SH Kim TK Choi HJ Lee SJ. Increased Frequency of Osteoporosis and Bmd Below the Expected Range for Age Among South Korean Women With Rheumatoid Arthritis. Int J Rheuma Dis (2012) 15(3):289–96. doi: 10.1111/j.1756-185X.2012.01729.x 22709491

[35] Kwon OC Oh JS Hong S Lee CK Yoo B Kim YG. Conventional Synthetic Disease-Modifying Antirheumatic Drugs and Bone Mineral Density in Rheumatoid Arthritis Patients With Osteoporosis: Possible Beneficial Effect of Leflunomide. Clin Exp Rheumatol (2019) 37(5):813–9. 30767868

[36] Clarke B. Normal Bone Anatomy and Physiology. Clin J Am Soc Nephrol (2008) 3 Suppl 3:S131–9. doi: 10.2215/CJN.04151206 PMC3152283 18988698

[37] Schipper LG Vermeer M Kuper HH Hoekstra MO Haagsma CJ Den Broeder AA. A Tight Control Treatment Strategy Aiming for Remission in Early Rheumatoid Arthritis Is More Effective Than Usual Care Treatment in Daily Clinical Practice: A Study of Two Cohorts in the Dutch Rheumatoid Arthritis Monitoring Registry. Ann Rheuma Dis (2012) 71(6):845–50. doi: 10.1136/annrheumdis-2011-200274 22210852

[38] Alemao E Joo S Kawabata H Al MJ Allison PD Rutten-van Mölken MP. Effects of Achieving Target Measures in Rheumatoid Arthritis on Functional Status, Quality of Life, and Resource Utilization: Analysis of Clinical Practice Data. Arthritis Care Res (2016) 68(3):308–17. doi: 10.1002/acr.22678 PMC5067571 26238974

[39] Saag KG Emkey R Schnitzer TJ Brown JP Hawkins F Goemaere S. Alendronate for the Prevention and Treatment of Glucocorticoid-Induced Osteoporosis. Glucocorticoid-Induced Osteoporosis Intervention Study Group. N Engl J Med (1998) 339(5):292–9. doi: 10.1056/NEJM199807303390502 9682041

[40] Cohen S Levy RM Keller M Boling E Emkey RD Greenwald M. Risedronate Therapy Prevents Corticosteroid-Induced Bone Loss: A Twelve-Month, Multicenter, Randomized, Double-Blind, Placebo-Controlled, Parallel-Group Study. Arthritis Rheum (1999) 42(11):2309–18. doi: 10.1002/1529-0131(199911)42:11<2309::AID-ANR8>3.0.CO;2-K 10555025

[41] Sambrook PN Cohen ML Eisman JA Pocock NA Champion GD Yeates MG. Effects of Low Dose Corticosteroids on Bone Mass in Rheumatoid Arthritis: A Longitudinal Study. Ann Rheuma Dis (1989) 48(7):535–8. doi: 10.1136/ard.48.7.535 PMC1003811 2774695

[42] Blavnsfeldt AG de Thurah A Thomsen MD Tarp S Langdahl B Hauge EM. The Effect of Glucocorticoids on Bone Mineral Density in Patients With Rheumatoid Arthritis: A Systematic Review and Meta-Analysis of Randomized, Controlled Trials. Bone (2018) 114:172–80. doi: 10.1016/j.bone.2018.06.008 29913256

[43] van der Goes MC Jacobs JW Jurgens MS Bakker MF van der Veen MJ van der Werf JH. Are Changes in Bone Mineral Density Different Between Groups of Early Rheumatoid Arthritis Patients Treated According to a Tight Control Strategy With or Without Prednisone If Osteoporosis Prophylaxis Is Applied? Osteo Internat: J Establish As Res Cooperat Between Eur Foundat Osteo Natl Osteo Foundat USA (2013) 24(4):1429–36. doi: 10.1007/s00198-012-2073-z PMC3604583 23011680

[44] Xie W Huang H Li G Hao Y Gui Y Wang Y. Dynamical Trajectory of Glucocorticoids Tapering and Discontinuation in Patients With Rheumatoid Arthritis Commencing Glucocorticoids With Csdmards: A Real-World Data From 2009 to 2020. Ann Rheuma Dis (2021) 2:annrheumdis-2021-220112. doi: 10.1136/annrheumdis-2021-220112 33811037

