# Supplementary material for: Impact of Treat-to-Target Therapy on Bone Mineral Density Loss in Patients With Rheumatoid Arthritis: A Prospective Cohort Study
Source: Front Endocrinol (Lausanne). 2022 May 17;13:867610. doi: 10.3389/fendo.2022.867610 (PMC9152020; doi:10.3389/fendo.2022.867610)

Supplementary Material

# Supplementary Table S1. Medications at baseline and during the first year and the first three years

| Drugs | During 1-year follow-up  n=144 | During 3-year follow-up  n=67 |
| --- | --- | --- |
| MTX, n (%) | 130 (90.3) | 60 (89.6) |
| LEF, n (%) | 44 (30.6) | 30 (44.8) |
| HCQ, n (%) | 76 (52.8) | 42 (62.7) |
| SASP, n (%) | 16 (11.1) | 14 (20.9) |
| Glucocorticoid, n (%) | 35 (24.3) | 15 (22.4) |
| Bisphosphonate, n (%) | 28 (19.4) | 24 (35.8) |
| Calcium supplements, n (%) | 100 (69.4) | 62 (92.5) |
| Vitamin D, n (%) | 69 (47.9) | 45 (67.2) |
| Biological agents, n (%) | 12 (8.3) | 4 (6.0) |

# Supplementary Table S2. Influence of various factors on %ΔBMD at lumbar spine during the first year: results of multiple linear regression analysis (N=144)

| Variables | β | 95% CI | *p* |
| --- | --- | --- | --- |
| Analysis with AMDAS28 |  |  |  |
| Female | -2.896 | -5.50, -0.29 | 0.030 |
| Bisphosphonate | 4.838 | 2.41, 7.27 | ＜0.001 |
| AMDAS28 | -1.107 | -2.17, -0.05 | 0.041 |
| Analysis with AMCDAI |  |  |  |
| Female | -3.002 | -5.51, -0.50 | 0.019 |
| Bisphosphonate | 4.710 | 2.34, 7.08 | ＜0.001 |
| AMCDAI | -0.317 | -0.52, -0.12 | 0.002 |

AMDAS28: adjusted-mean disease activity scores based on erythrocyte sedimentation rate; AMCDAI adjusted-mean clinical disease activity index

# Supplementary Figure S1. Trends in the percentage of patients achieving remission, low, moderate, and high disease activity over 3 years by DAS28 (A), CDAI (B) and SDAI (C)

#
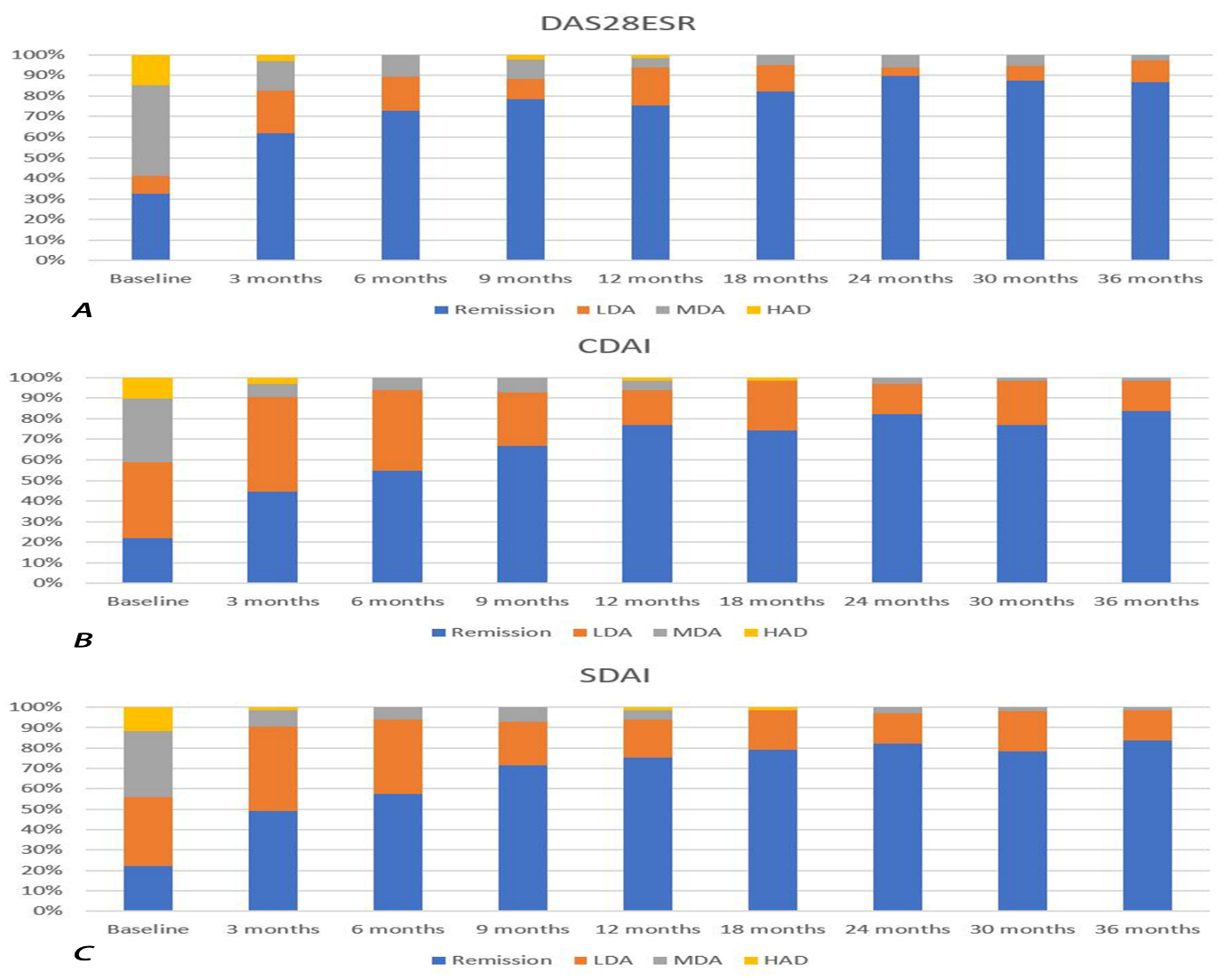

Supplement: Supplementary file 1 [file DataSheet_1.docx]
